# Visceral Leishmaniasis in Southwestern Iran: A Retrospective Clinico-Hematological Analysis of 380 Consecutive Hospitalized Cases (1999–2014)

**DOI:** 10.1371/journal.pone.0150406

**Published:** 2016-03-04

**Authors:** Bahador Sarkari, Tahereh Naraki, Mohammad Amin Ghatee, Samaneh Abdolahi Khabisi, Mohammad Hassan Davami

**Affiliations:** 1 Department of Parasitology and Mycology, School of Medicine, Shiraz University of Medical Sciences, Shiraz, Iran; 2 Basic Sciences in Infectious Diseases Research Center, Shiraz University of Medical Sciences, Shiraz, Iran; 3 Department of Parasitology and Mycology, School of Medicine, Jahrom University of Medical Sciences, Jahrom, Iran; University of Thessaly, Faculty of Medicine, GREECE

## Abstract

Visceral Leishmaniasis (VL) is an endemic parasitic disease and remains as a major health concern in southwestern Iran. The current study describes clinico-hematological, epidemiological and therapeutic features of VL cases, admitted to university-affiliated hospitals, during 1999–2014 in Fars province, southwestern Iran. A total of 380 VL cases were recorded during a 16 years period, giving an average annual admission of 23.75 cases/year in which 217 (57.1%) were male and 163 (42.9%) were female. Mean age of the patients was 3.7 years. The majority of the cases (91.5%) were ≤ 5 years old. Bone-marrow aspiration detected *Leishmania* amastigotes only in 26.6% of cases. Fever (98.1%), abdominal protrusion (65.1%) and hepatosplenomegaly (63.7%) were the most common clinical presentations of the patients. Pancytopenia was noted in 43.1, anemia in 87.3 and thrombocytopenia in 64% of cases. Increase in the level of AST (aspartate aminotransferase), ALT (alanine aminotransferase), alkaline phosphatase, LDH (lactate dehydrogenase) and CRP (C-Reactive Proteins) were seen in 84.9, 53.6, 44.4, 72.5 and 83.1% of cases, respectively. Mortality was noted in 5.3% of cases. Deranged haemato-biochemical parameters including total and direct bilirubin, PLT (platelet) and pancytopenia were significantly contributed to mortality from VL. Moreover, clinical features such as severe splenomegaly as well as bacterial infections were meaningfully contributed to death from VL. The majority of patients (74.9%) were treated with meglumine antimoniate. Amphotericin B was administrated in 59 of cases, 11 of them were initially treated with meglumine antimoniate with a shift to amphotericin B, because of treatment failure. Findings of the current study demonstrated that VL is present in southwest of Iran with a fairly continual rate during the last 16 years period. Deranged haemato-biochemical parameters along with severe splenomegaly contributed to mortality from VL.

## Introduction

Leishmaniasis is an important and neglected parasitic disease in the world. Cutaneous and visceral leishmaniasis are major health problems in the Eastern Mediterranean Region (EMR) of WHO where cutaneous leishmaniasis (CL) and visceral leishmaniasis (VL) are seen in 14 out of 22 countries of the region [1]. VL caused by *L*. *infantum* occurs in most of the countries in that region, apart from Sudan and Somalia, where VL is caused by *L*. *donovani* [1]. Both CL and VL are present in Iran [2–4]. VL is endemic in few provinces of Iran, including Ardabil (northwest), Fars (southwest), East Azarbayjan (East), Chaharmahal and Bakhtiari (southwest), Bushehr (the coastal region on the Persian Gulf) and Khuzestan (south) [2]. During the last decades, more than 2000 cases of VL have been reported from 31 Iranian provinces, with about 100 to 300 cases annually. From these, 44.6% were reported from northwestern Iran. The average annual number of diagnosed VL cases in Iran has been 0.449 cases/100,000 inhabitants during the last decade [2, 5]. Dogs are considered as the main reservoir of the infection, although infections in other animals such as rodents and cats have been documented [6–8].

VL in Iran is commonly caused by *L*. *infantum* with children younger than 5 years old considered as the main victim of the disease [2, 9]. Diagnosis of VL is mainly based on detection of amastigotes of *Leishmania* in bone marrow aspirate or liver or spleen biopsies. However, these approaches are invasive and antibody detection methods, including Indirect Fluorescent Antibody Test (IFAT), Enzyme-linked Immunosorbent Assay (ELISA) and Direct Agglutination Test (DAT), or antigen detection methods such as latex agglutination test are being used nowadays for diagnosis of VL [10–14].

Fever is the most common manifestation of VL, which may last for few weeks, and hepatomegaly and splenomegaly are cardinal features of the disease. Clinical and laboratory findings of VL may be different in VL patients in different geographical areas based on the causative agents of the disease. In the current study the clinico-hematological, epidemiological and therapeutic features of VL cases, admitted to university-affiliated hospitals during a 16 years period in Fars province, southwestern Iran, were retrospectively analyzed.

## Materials and Methods

The study was approved by Ethic Committee of Shiraz University of Medical Sciences and patients’ record were anonymized and de-identified prior to analysis. Clinical and laboratory parameters along with demographic data of VL patients admitted to university-affiliated hospitals during 1999–2014 (16 years) in Fars province, southwest of Iran, were evaluated. These hospitals act as referral centers for all of southern part of the country.

The study area, Fars province, is one of the 31 provinces in Iran, located in southwest of the country. Fars is one of the main foci of both CL and VL in Iran. Cases of VL also reported from the neighboring provinces, Bushehr and Kohgiluyeh and Boyer-Ahmad and such cases are also included in this study, since they admitted to the hospitals in Shiraz, capital of Fars province, as tertiary referral hospitals.

VL diagnosis was based on microscopic demonstration of *Leishmania* amastigotes on Giemsa stained smears of bone-marrow aspiration or spleen biopsy, serology (mainly IFAT), and *ex Juvantibus*. Patients with negative bone-marrow or negative or borderline serology, but with persisting clinical and laboratory features suggesting of VL, and responding to the VL treatment, were also considered as VL and recorded as VL patients in the hospital records. The VL patients had specific hospital record number and patients’ data were retrieved from their medical records.

### Statistical analysis

Data were collected in a data sheet and analyzed by SPSS Ver. 22 (SPSS Inc., Chicago, IL, USA). Chi-square was used to determine the association of VL disease and demographic or clinico-hematological values by comparing the proportions of any variable in different groups. For analysis of hematological records, normal values for each parameter were considered and patient’s records were compared with those values.

## Results

A total of 380 VL cases were recorded in 1999–2014 (16 years), giving an average annual admission rate of 23.75 cases/year.

### Epidemiological features of the VL patients

Mean age of the patients was 3.7 years (±8.1 years). Median age of the patients was 1.3 years (range, 1 month to 75 years). The majority of the cases (91.5%) were ≤ 5 years and 94% were ≤ 10 years old. From 380 VL cases, 217 (57.1%) were male and 163 (42.9%) were female. Cases were from 42 different peripheral districts in the province. Most cases were from Darab (42 cases, 11.1%) followed by Kazeroun (37 cases, 9.7%) and Firoozabad (31 cases 8.2%).

Most of VL cases were in year 2006 (42 cases, 11.1%) followed by 2003 (36 cases, 9.5%), 2004 and 2005 (35 cases, 9.2%, in each year). Considering the monthly distribution of VL cases, based on the date of admission, patients were admitted throughout the year with most cases in January (53 cases, 13.9%), February (47 cases, 12.4%) and March (45 cases, 11.8%) and the least cases in August (19 cases, 5%), September (21 cases, 5.5%), and November (21 cases, 5.5%).

### Clinical features of the VL patients

VL diagnosis has been based on clinical signs and symptoms plus positive serological (IFAT) or parasitological tests. IFAT have been positive (titer of ≥1/128) in 69.1%, weak (titer of 1/64) in 20.1% and negative in 10.9% of cases. Bone-marrow aspiration has been performed in 241 cases and *Leishmania* amastigotes were detected only in 64 (26.6%) of cases.

Fever (98.1%), abdominal protrusion (65.1%) and hepatosplenomegaly (63.7%) were the most common clinical presentations of the disease. Splenomegaly (without hepatomegaly) and hepatomegaly (without splenomegaly) were seen in 23.7 and 2.5% of the cases, respectively. Sever splenomegaly, as defined by Poulin et al., (1998), or hepatomegaly was seen in 5.2 and 2.2% of the cases, respectively [15].

Other noticeable clinical features of the patients were chilling (16%), sweating (9.6%), lethargy (17.6%), anorexia (24.3%), vomiting (17.6%), diarrhea (17.4%), poor feeding (22.9%), weight loss (13.6%), cough (17.9%) jaundice (11.7%), edema (2.4%), dyspnea (2.7%), and convulsion (1.1%). Concurrent bacterial infections were seen in most of cases. These cases were treated with different antibiotics including, Ceftriaxone (29.7%), Cefixime (0.8%), Gentamicin (9.6%), Ampicillin (10%), Ceftazidime (4.2%), Rifampin (1.3%), Amikacin (3.3%), Vancomycin (7.5%). Nystatin has also been used in 2.5% of cases for treatment of fungal infections.

The most commonly used organic compounds of antimony (Sb) in this study have been meglumine antimoniate (Glucantime). The majority of patients (74.9%) were treated with meglumine antimoniate (mean dose 20 mg antimony/kg/day). Duration of treatment was 28 days. A course of 12-day treatment has also been used in few cases with equal efficacy [16]. Amphotericin B (1.0 mg/kg/day for 15 days or 1.0 mg/kg/every other day for 30 days) has been used in 59 of cases. Out of these 59 cases, 11 cases were initially treated with meglumine antimoniate with a shift to amphotericin B because of unresponsiveness to the pentavalent antimonial drugs. Defervescence and clinical improvement have been considered as responsiveness to treatment. Relapse, defined as reappearance of signs and symptoms of disease after successful initial treatment, was seen in 5.8% of cases. Amphotericin B was used in the second course of treatment in 66.6% of the cases. The adverse effect of the drug treatment has been recorded in some cases which included, abdominal pain, anorexia, vomiting, nausea, myalgia, arthralgia, headache, and malaise. Impairment of liver or kidney functions has also been recorded in a few cases.

### Biochemical and hematological features of the VL patients

Laboratory findings revealed anemia in 87.3% of cases. Pancytopenia was noted in 43.1% of cases. Platelet counts were recorded in 364 cases, among them thrombocytopenia was observed in 64% and thrombocytosis in 1.9% of the cases. White blood cells (WBC) counts were recorded in 372 cases, among them 39.5% had high and 7.5% had low WBC count.

Urine analysis was abnormal in 37.7% of cases and urine cultures were positive in 28.3% of the cases. Blood culture was recorded for 108 cases, among them 11.1% had positive blood culture. Fecal Occult blood (OB) was positive in 14.6% of cases. AST, ALT and alkaline phosphatase values were abnormal (high) in 84.9, 53.6 and 44.4% of cases, respectively.

Decreased albumin/globulin ratio was noted in 46% of cases. Triglyceride was recorded only for 34 cases; among them the value level was higher than normal in 94.1% of cases. LDH was higher than normal value in 72.5% of cases. Increased ESR, CRP and PPT levels were seen in 53.2, 83.1 and 57.5% of cases, respectively. Hematological and biochemical features of VL patients in this study are summarized in Table 1.

**Table 1:** Hematological and biochemical features of VL patients.

Parameters	No. of patients analyzed*		Patients with normal values		Patients with abnormal values	
	No.	percent	No.	Percent	No.	Percent
U/A	159	41.8	99	62.2	60	37.7
U/C	138	36.3	99	71.7	39	28.3
B/C	108	28.4	96	88.9	12	11.1
OB	96	25.3	82	85.4	14	14.6
WBC	372	97.9	197	53	175	47
Hb	364	95.8	59	16.2	305	83.8
PLT	360	94.7	40	11.1	320	88.8
AST	219	57.6	33	15.1	186	84.9
ALT	220	57.9	102	46.4	118	53.6
Alb	239	62.9	139	58.2	100	41.8
ALb/Glu	339	89.2	129	54	110	46
ALKP	207	54.5	115	55.6	92	44.4
Total bilirubin	205	53.9	156	76.1	49	23.9
Directbilirubin	207	54.5	162	78.3	45	21.7
BUN	198	52.1	191	96.5	7	3.5
Cr	175	46.1	172	98.3	3	1.7
Na	182	47.9	132	72.5	50	27.5
K	185	48.7	136	73.5	49	18.3
BS	136	35.8	107	78.6	29	21.3
Ca	112	29.5	86	76.7	26	23.3
P	35	9.2	13	37.1	22	62.9
TG	34	8.9	2	5.89	32	94.1
CHOL	31	8.2	19	61.3	12	38.7
LDH	80	21.1	22	27.5	58	72.5
ESR	154	40.5	72	46.8	82	53.2
CRP	154	40.5	26	16.9	128	83.1
PT	128	33.7	95	74.2	33	25.8
PTT	113	29.7	48	42.5	65	57.5

U/A: urine analysis; U/C: urine culture; B/C: blood culture; OB: occult blood; WBC: white blood cells; Hb: hemoglobin; PLT: platelet; AST: aspartate aminotransferase; ALT: alanine aminotransferase; Alb: albumin; Alb/Glu: albumin/globulin ratio; ALKP: alkaline phosphatase; BUN: blood urea nitrogen; Cr: creatinine; BS: blood sugar; TG: triglyceride; CHOL: cholesterol; LDH: lactate dehydrogenase; ESR: erythrocyte sedimentation rate; CRP: C-reactive protein; PT: prothrombin time; PTT: partial thromboplastin time.

*: The test has not been requested for the rest of the patients.

Mortality rate of VL patients was 5.3% in the current study. Chi-square was used to determine associations between mortality due to VL and clinic-hematological or demographical features of the patients. Higher mortality rate was seen in males than females but the difference was not statistically significant (P>0.05). Significant association were found between death from VL and deranged haemato-biochemical parameters including total bilirubin (*x*^*2*^ = 15.23, *df* = 1, P = 0.001) direct bilirubin (*x*^*2*^ = 17.75, *df* = 1, P = 0.001), WBC count (*x*^*2*^ = 14.53, *df* = 2, P = 0.001) pancytopenia (*x*^*2*^ = 7.6, *df* = 1, P = 0.006) and also clinical features such as severe splenomegaly (*x*^*2*^ = 5.37, *df* = 2, P = 0.041). Mortality rate was more common in relapse cases as well as in cases with bacterial infections.

## Discussion

VL is still a health problem in Iran and cases of VL are frequently reported from different areas, mainly from the southwest and northwest of the country [2, 5]. In the current study clinical and hematological features of 380 VL cases, admitted consecutively to hospitals during a 16 years period in southwestern Iran, were retrospectively analyzed.

VL is characterized by irregular fever, enlargement of spleen and liver, progressive weakness which may result in death, if left untreated. Moreover, VL can cause a variety of hematological disorders such as leukopenia, thrombocytopenia, pancytopenia and anemia.

Petrela et al., reviewed 1210 cases of VL in Albania and found hepatosplenomegaly in 100, fever in 95.4 and anemia in 88% of the cases [17]. Retrospective evaluation of 111 VL cases in Italy revealed fever and splenomegaly in 100, hepatomegaly in 90 and anemia in 70.3% of the cases [18]. Dominant signs and symptoms in VL patients in a series of 217 hospitalized cases in northwest of Iran were paleness, (99.5%), fever (96.9%), splenomegaly (91.5%), hepatomegaly (53.6%) and lymphadenopathy (21.2%) [2].

The causes of anemia in VL are linked to sequestration and destruction of red blood cells (RBC) in enlarged spleen, immune mechanisms and also alteration in RBC membrane permeability [19].

Deranged hematological and biochemical parameters is another feature of VL. Increase in the level of lactate dehydrogenase, triglyceride, AST and CRP were seen in VL patients evaluated in this study. Moreover, pancytopenia was noted in 43.1, anemia in 87.3 and thrombocytopenia in 64% of VL cases. In agreement with our results, analysis of hematological parameters of VL patients in Italy revealed thrombocytopenia and anemia, both in 80.4 and leukopenia in 43.1% of the patients [18]. In a retrospective study of 546 children with VL from Brazil by Sampaio et al., the presence of severe neutropenia and severe thrombocytopenia were reported as independent predictors of death from VL. In their study severe thrombocytopenia had the strongest association with death [20].

VL in Iran is a pediatric infection and the main victims of the disease are children under 10 years old. It has been reported that up to 99% of VL cases in Iran are under 12 years old [2]. From 246 cases of VL in northwestern Iran, 91% were ≤5 years old [2]. In a series of 1210 cases of VL from Albania, all the patients have been in the age range of 0–14 years, with a median of 4 years [17]. In our study, only small number of cases of VL (3 cases) were aged higher than 40 years. It is worth mentioning that the causative agent of few immunocompetent VL cases, aged higher than 40 years, in Iran has been *L*. *tropica* rather than *L*. *infantum (*unpublished data). However, the causative agent of VL in older people has not been determined in the current study.

VL is a serious parasitic disease and is fatal if left untreated. Mortality associated with VL in series of 602 hospitalized cases from northwestern Iran was 2.8% [2]. In the current study a mortality rate of 5.3% was noted.

In a review of 546 VL cases by Sampaio et al., in Brazil, 57 deaths were reported. The main clinical mortality risk factors was from mucosal bleeding, jaundice, bacterial infections and dyspnea and the main laboratory risk factors consisted of neutrophil count of <500/mm^3^ and platelet count of <50,000/ mm^3^. Authors proposed these factors as predictors of risk of dying from VL [19]. In Sudanese VL patients, risk factor for death were identified as, age less than 2 years, malnutrition, anemia, hemoglobin <6 g/dl, and splenomegaly [21].

Bacterial superinfection is one of the major complications leading to death in VL patients [22]. In infants with visceral leishmaniasis, fatal bacterial infections can be accompanied by nonspecific signs and symptoms [23]. Administration of broad-spectrum antibiotic treatment in all patients under one year old has been a part of VL treatment in this study [23].

In the current study the majority of VL cases were detected during January to April. This is consistent with previous studies in Iran which reported the occurrence of most of VL cases during January up to July [2]. These are linked to the period of sandfly activities which usually start in August, and the average incubation period of VL, which is about 3–6 months.

In the current study sensitivity of bone-marrow microscopy evaluation for detection of *Leishmania* amastigotes was relatively low as parasites were detected in only 26.6% of the bone marrow samples. Bone marrow aspirations have been positive in most (77.5%) of VL patients from Albania and also from Sicily (80.2%) in Italy [17, 18]. The sensitivity of bone marrow aspiration for diagnosis of VL is low (50% to 85%) in comparison with spleen aspiration (93%–98.7%) [24]. In three studies, carried out in Shiraz, Iran, bone marrow aspiration were positive in only 35–50% of symptomatic VL cases while 89.5% of the splenic smears were found to be positive for *Leishmania* amastigotes [23]. In those previous studies, spleen puncture resulted in severe hemorrhage and hypotension in two patients which necessitated splenectomy in one case [23]. Nowadays, physicians in the area rely mainly on serological tests along with clinical signs and symptoms for the diagnosis of VL. The strain of parasite and parasite’s load in bone marrow might have contributed to the low sensitivity of bone marrow aspiration in our region for the diagnosis of VL. Parasite load in bone marrow aspirate of VL patients in the region was shown to be low, as results of culture in NNN media have been disappointing in few studies in this area [23].

Pentavalent antimonial are still the drug of choice for VL treatment, because of their low cost, availability and suitable efficacy. In the current study, a 28-day course of meglumine antimoniate (Glucantime) has been used in most cases. However, a shortened course of treatment (12-day) has been used in some cases with equal efficacy. This includes a single deep intramuscular injection of 20 mg/kg/day Glucantime continued for seven days after defervescence [16].

Resistance to pentavalent antimonial has been documented in CL patients in Iran [25, 26]. Nevertheless, such resistance has not been reported in VL cases in the country. A few cases of unresponsiveness to meglumine antimoniate treatment (11 cases) were seen in this study. These patients were subsequently treated successfully with amphotericin B.

In-built to any retrospective studies, recording of the cases of VL and also clinical and laboratory features of VL patients might not been fully appropriate in this study. This is because there is no registry system for VL in Iran and some VL cases might not been properly recorded. Moreover, a few VL cases might have been treated in peripheral hospitals, especially during the recent years that hospitals in peripheral districts have been equipped to treat such cases.

Taken together, findings of the current study demonstrated that VL is present in southwest of Iran with a relatively constant rate during the last 16 years period of evaluation. Deranged haemato-biochemical parameters along with severe splenomegaly as well as bacterial infections contributed to mortality from VL.

## Supporting Information

S1 Table SPSS data of the VL patients. (SAV)
